# Defining distribution and habitat use of west‐central Florida’s coastal sharks through a research and education program

**DOI:** 10.1002/ece3.8277

**Published:** 2021-10-26

**Authors:** Lindsay L. Mullins, J. Marcus Drymon, Moriah Moore, Adam Skarke, Alan Moore, John C. Rodgers

**Affiliations:** ^1^ Coastal Research and Extension Center Mississippi State University Biloxi Mississippi USA; ^2^ Department of Geosciences Mississippi State University Mississippi State Mississippi USA; ^3^ Northern Gulf Institute Starkville Mississippi USA; ^4^ Mississippi‐Alabama Sea Grant Consortium Ocean Springs Mississippi USA; ^5^ Coastal Marine and Education Research Academy Clearwater Florida USA; ^6^ Texas Parks and Wildlife Dickinson Texas USA

**Keywords:** coastal, elasmobranch, habitat use, non‐traditional data

## Abstract

Identifying critical habitat for highly mobile species such as sharks is difficult, but essential for effective management and conservation. In regions where baseline data are lacking, non‐traditional data sources have the potential to increase observational capacity for species distribution and habitat studies. In this study, a research and education organization conducted a 5‐year (2013–2018) survey of shark populations in the coastal waters of west‐central Florida, an area where a diverse shark assemblage has been observed but no formal population analyses have been conducted. The objectives of this study were to use boosted regression tree (BRT) modeling to quantify environmental factors impacting the distribution of the shark assemblage, create species distribution maps from the model outputs, and identify spatially explicit hot spots of high shark abundance. A total of 1036 sharks were captured, encompassing eleven species. Abundance hot spots for four species and for immature sharks (collectively) were most often located in areas designated as “No Internal Combustion Engine” zones and seagrass bottom cover, suggesting these environments may be fostering more diverse and abundant populations. The BRT models were fitted for immature sharks and five species where *n* > 100: the nurse shark (*Ginglymostoma cirratum*), blacktip shark (*Carcharhinus limbatus*), blacknose shark (*C. acronotus*), Atlantic sharpnose shark (*Rhizoprionodon terraenovae*), and bonnethead (*Sphyrna tiburo*). Capture data were paired with environmental variables: depth (m), sea surface temperature (°C), surface, middle, and bottom salinity (psu), dissolved oxygen (mg/L), and bottom type (seagrass, artificial reef, or sand). Depth, temperature, and bottom type were most frequently identified as predictors with the greatest marginal effect on shark distribution, underscoring the importance of nearshore seagrass and barrier island habitats to the shark assemblage in this region. This approach demonstrates the potential contribution of unconventional science to effective management and conservation of coastal sharks.

## Graphical abstract

Identifying critical habitat for highly mobile species such as sharks is difficult, but essential for effective management and conservation. Using shark capture data collected by a research and education group in Florida, this study used boosted regression tree (BRT) modeling to quantify environmental factors impacting the distribution of the shark assemblage, create species distribution maps from the model outputs, and identify spatially explicit hot spots of high shark abundance. This approach for identifying spatially explicit areas and environmental conditions suited for shark abundance demonstrates the potential contribution of unconventional science to effective management and conservation of coastal sharks.

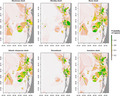

## 1 INTRODUCTION

Sharks are common mid‐to‐upper‐level marine predators that contribute to the health of the world's oceans by influencing marine species populations through predator–prey interactions (Heithaus et al., 2002; Simpfendorfer et al., 2001). They help to balance coastal and marine ecosystems by regulating them vertically, horizontally, and temporally through predation and intimidation, which can support healthier prey populations and mitigate overgrazing of ecologically foundational seagrass meadows (Dulvy et al., 2017; Heithaus et al., 2007). Compared to many fish, sharks are generally late maturing with low reproductive output, rendering the survival of juvenile individuals critical to the success of a population (Kindsvater et al., 2016). As a result, efforts to identify juvenile shark habitat and understand the environmental factors that make it suitable, particularly in the face of a changing climate (Dulvy et al., 2017), are critical to shark management efforts.

Like many fish species, sharks often use coastal and estuarine areas as nurseries for juveniles due to their elevated levels of productivity, shallow protected waters, and high abundance of prey (Beck et al., 2001). Heupel et al. (2007) have expanded upon these characteristics to establish criteria specific to elasmobranchs, defining shark nurseries as locations where (1) relative abundance of sharks is greater on average than over all areas; (2) sharks exhibit site fidelity, returning or remaining in the area for extended periods of time; and (3) the area is used repeatedly across years. In addition to providing critical juvenile shark habitat, high shark species diversity has been found in these habitats surrounding barrier islands and around river mouths of the Gulf of Mexico (Bethea et al., 2014). Despite the instrumental role of these habitats in sustaining healthy shark populations, many potential nursery areas lack baseline population data.

In Florida, spatially explicit shark distribution data have been used to further understand species life cycles and consequently, to directly inform conservation practices in estuaries. Evaluation of these data has suggested that parameters such as temperature and salinity drive species distribution, as well as influence size‐based habitat partitioning. For example, salinity and temperature have been shown to determine size partitioning of bull sharks (*Carcharhinus leucas*) across estuarine habitats in Florida (Simpfendorfer et al., 2005). Further, Brooks et al. (2019) reviewed the capacity of spatial delineation of habitat in the implementation of successful fisheries conservation strategies. When an aggregation of breeding lemon sharks (*Negaprion brevirostris*) was identified in a nearshore Floridian estuarine environment, efficient communication between scientists and stakeholders coupled with the availability of spatially explicit data was used to successfully designate the area as a Habitat Areas of Particular Concern (HAPC) by NOAA Fisheries. As human development continues to increase along Florida's coastline, further efforts to implement conservation strategies based on spatially explicit data are urgently needed for nearshore aquatic habitats.

Two types of data are commonly used in fisheries management and conservation: fisheries‐dependent (i.e., data collected by fishermen) or fisheries‐independent (i.e., data collected by professional scientific researchers, usually affiliated with academia or government organizations). However, some protected areas lack either type of baseline population data, which, in turn, limits our understanding of the ecosystem and the effectiveness of conservation strategies that are implemented (Ward‐Paige & Worm, 2017). Data collected by third parties, such as citizen scientists and private research and education groups, provide an alternate form of fisheries‐independent data. These can be used to understand species distribution and develop spatially explicit management and conservation strategies, an approach that has been successfully implemented in other scientific disciplines such as ornithology and astronomy (Dickinson et al., 2010). While there are concerns over the accuracy of data collected by non‐professionals, older volunteers with college experience and those accompanied by professionals demonstrate increased accuracy in scientific performance (Dickinson et al., 2010). In addition, there is often a lack of available funding for traditional fisheries‐independent data collection, but citizen science groups may require payment from their participants to fund the research (Dickinson et al., 2010). The Coastal Marine and Education Research Academy (CMERA), located in Pinellas County, Florida, is an example of a research and education group. Undergraduate and graduate students pay to participate in the program to learn about sharks and rays and gain experience with professional scientists sampling in the field. These efforts have resulted in the dataset used in this study, which details shark and ray captures along the west‐central Florida coast since the organization's establishment in 2013.

The coastal waters of west‐central Florida contain a variety of bay, estuarine, and barrier island habitats. A great diversity of sharks with respect to species and maturity has been noted by CMERA in these habitats; however, a formal population study has never been conducted there, and explicit habitat use of sharks in that region remains unknown. Given the available data and the potential for this area to function as critical habitat, such as a nursery area, there is a need for baseline population analyses and an understanding of factors driving shark distribution. The purpose of this study is to examine the utility of data collected by research and education programs to identify areas of clustering, determine which environmental parameters may be driving shark distribution, and to create spatially explicit distribution maps for selected species and immature sharks in the coastal waters of west‐central Florida.

## 2 METHODS

### 2.1 Study area

**FIGURE 1:**
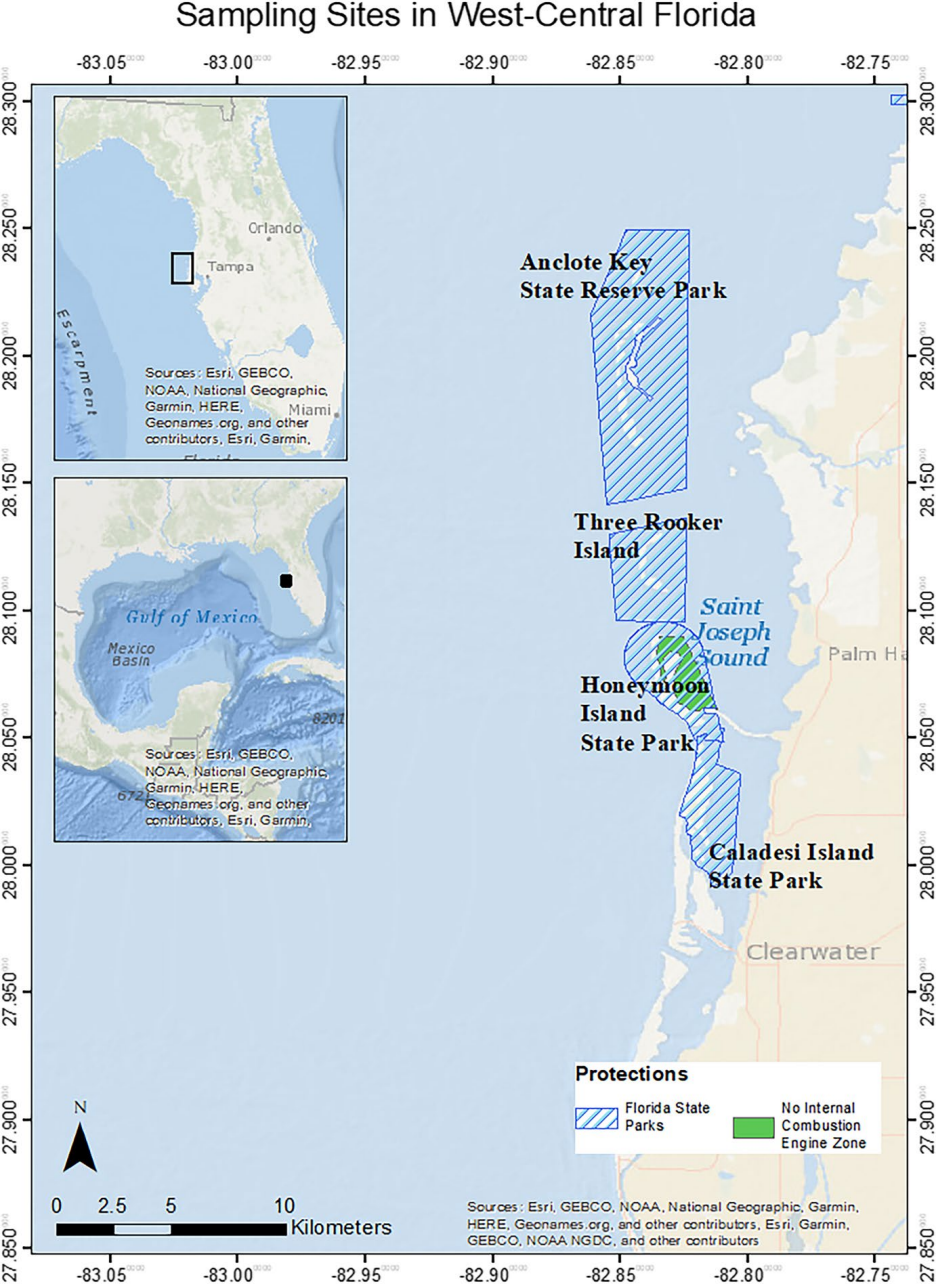
Study area in west‐central Florida. Inset map denotes geographic range of the study area within the context of Florida and the Gulf of Mexico. Florida State Parks are indicated by dashed blue lines and the area protected as a No Internal Combustion Engine Zone by green polygon

Data were collected along the west‐central Florida coast in waters adjacent to Pinellas County, primarily within the Gulf Intracoastal Waterway. The study area encompasses several barrier islands, many of which are protected under the state park system. The waters east of Honeymoon Island State Park have additional protection as a “No Internal Combustion Engine” (NICE) zone. Sites were initially selected haphazardly due to limitations such as boat and fishing accessibility, and remained fixed across sampling years. During sampling season, sites were comprehensively sampled within two‐week time frames. The majority of CMERA study sites are within seven miles of the coast to the east and are bordered by barrier islands to the west (Figure 1). A subset of sites lie further west outside of the barrier islands as far as 15 miles from the coast.

St. Joseph Sound is separated from the Gulf of Mexico by barrier islands to the east. The bays range from 30 to 600 m in width and are connected to the Gulf of Mexico through a series of inlets separating the barrier islands, which provide a pathway for shark movement between the ocean and the nearshore bays and estuaries. Overall, this area is considered low energy, characterized by infrequent hurricanes and mild winter frontal systems. Longshore sediment transport along the coast is driven south to north by prevailing wave conditions. Prior to 1950, much of the shorelines of the barrier islands was receding. Since then, management efforts such as beach renourishment projects as well as the construction of groins, jetties, and seawalls have created regions of accretion and shoreline advance (PCPWD, 2017).

### 2.2 Fieldwork

Fieldwork was conducted by CMERA from 2013 to 2018 during the months of May–August each year. A total of 47 sites were regularly sampled and were classified according to their depth and bottom type. Sampling was conducted by college students under the direct training and supervision of CMERA staff, who also provided quality control of data. Individual sharks were captured using longline, tangle net, and rod and reel methods. Longlines were set for 45 min and included a 152.4 m mainline, 30 gangions with 20/0 hooks baited with cut mullet (*Mugil cephalus*), and a buoy and anchor attached at the ends. Tangle nets were set for 30 min and were made of nylon, with a buoyant float line and leaded bottom line. Tangle nets were 61 m long, 2 m high, and had a 10 cm stretch mesh. For each individual capture, the following data were collected: longitude and latitude (World Geodetic System (WGS), 1984), depth (m), bottom type (sand, seagrass, or artificial reef), water temperature (°C), tidal stage, species, sex, pre‐caudal length, fork length, and total length (cm) and other details such as noticeable wounds or external tags. For male individuals, maturity was determined by CMERA according to clasper calcification (Clark & von Schmidt, 1965). Unless previously tagged, all sharks were tagged with FLOY FH‐69 tags.

### 2.3 Environmental parameters

Additional environmental data including salinity (psu), dissolved oxygen content (DO, mg/L), and seagrass extent were provided by Pinellas County Department of Environmental Management (PCDEM). Salinity, which was recorded as surface, middle, and bottom, and DO data were filtered to match the range of CMERA sampling dates and averaged across that range to create data points that reflected mean summertime values from 2013 to 2018. Seagrass extent was measured by Pinellas County in partnership with the Tampa Bay Estuary Program and the Southwest Florida Water Management District through transect analysis and bi‐annual aerial seagrass imagery and analysis, respectively. Additional synoptic surveys are conducted by Pinellas County in Clearwater Harbor and St. Joseph Sound. Water quality data were collected as part of routine sampling by PCDEM. They currently conduct eight water sampling periods per year in which seventeen sites are sampled. These periods consist of seven predetermined land‐accessible sites and at least eight randomly preselected boat‐accessible sites (PCDEM, 2017).

### 2.4 Data analysis

The sex ratio of the shark assemblage was examined by year, then assessed for statistical significance using a two‐sample *t* test assuming unequal variances. All spatial data were imported into a Geographic Information System (GIS; ESRI). A bottom‐type layer was created by combining the retrieved PCDEM seagrass layer and the reef locations according to CMERA’s notes, which were then corroborated with artificial reef coordinates available on the PCDEM website. Areas surrounding seagrass and reef locations were designated as “sand” bottom type. Data collected from PCDEM were filtered to match CMERA sampling dates and averaged at a location across the summer season. Environmental parameters were then interpolated using the inverse distance weighted (IDW) tool to create a continuous raster layer. Following interpolation, the study area covered approximately 36 × 25 km. Output resolution of raster files was 220 × 220 m. This resolution was determined by measuring the width of the narrowest site (site 30, approximately 220 m). This is the coarsest resolution which will allow this site to keep its approximate shape when rasterized. Given the relatively fine scale, we assume that variation of environmental data within a 220 × 220 m pixel will be minimal.

Catch‐per‐unit‐effort (CPUE) for each species (where *n* > 10) and for immature sharks collectively was calculated for each site by gear type (i.e., tangle nets or hooks), resulting in two CPUEs for each species by site, using the equation below:

$$\text{CPUE}=\frac{\text{number}\;\text{of}\;\text{captures}}{\text{gear}\;\text{type}\times \text{hr}\;\text{soaked}}$$

These CPUE values were then linked in the attribute table of sites. Next, a hot spot analysis (Getis‐Ord Gi*) was used to identify which sites may be experiencing spatial clustering for a particular shark species or age group. For each species and age group, boosted regression tree (BRT) models were created from 2013 to 2018 data according to methods set forth in Hijmans and Elith (2017) and Elith and Leathwick (2017). Specifically, environmental data were organized and validated according to Hijmans and Elith (2017), and model testing and species distribution map (SDM) creation followed Elith and Leathwick (2017). Only subgroups of the sampled population with sufficiently robust counts (*n* ≥ 100) were included (Pearson, 2010). These subgroups were as follows: immature sharks, nurse shark, blacktip shark, blacknose shark, Atlantic sharpnose shark, and bonnethead. Environmental variables considered in this analysis were water depth (m), sea surface temperature (SST, ℃), surface, middle and bottom salinity (psu), bottom type (sand, seagrass, or reef), and dissolved oxygen (DO) (mg/L). Due to the relatively small sample sizes and use of presence‐only data, distributions were modeled using Bernoulli BRTs. The use of Bernoulli BRTs also accounts for differences in method of capture, where each capture is presumed to mark suitable habitat. The BRTs were created to optimize a combination of parameters: learning rate (lr), bag fraction (bf), and tree complexity (tc). Based on a maximized cross‐validation area under the receiver operating curve (CV AUC), minimized standard error, and a maximized training data AUC (TD AUC), a specific combination of the aforementioned parameters was identified as the best fit (Hijmans & Elith, 2017). The ideal model resulting from this optimization was applied to the rasterized environmental data layers, then used to create the SDMs. The SDMs were then exported into ArcGIS. To be consistent with the resolution of the input environmental data, output resolution of SDMs was 220 × 220 m. Given that environmental conditions are unlikely to vary significantly within this pixel size, it is also unlikely that habitat suitability would vary significantly. A BRT model and a SDM were created for each of the aforementioned relevant shark subgroups. The BRT models were constructed in R version 2.6.2 (R Core Team, 2019) using the “gbm” package (v2.1.5, Greenwell et al., 2019).

## 3 RESULTS

### 3.1 CMERA survey data

**FIGURE 2:**
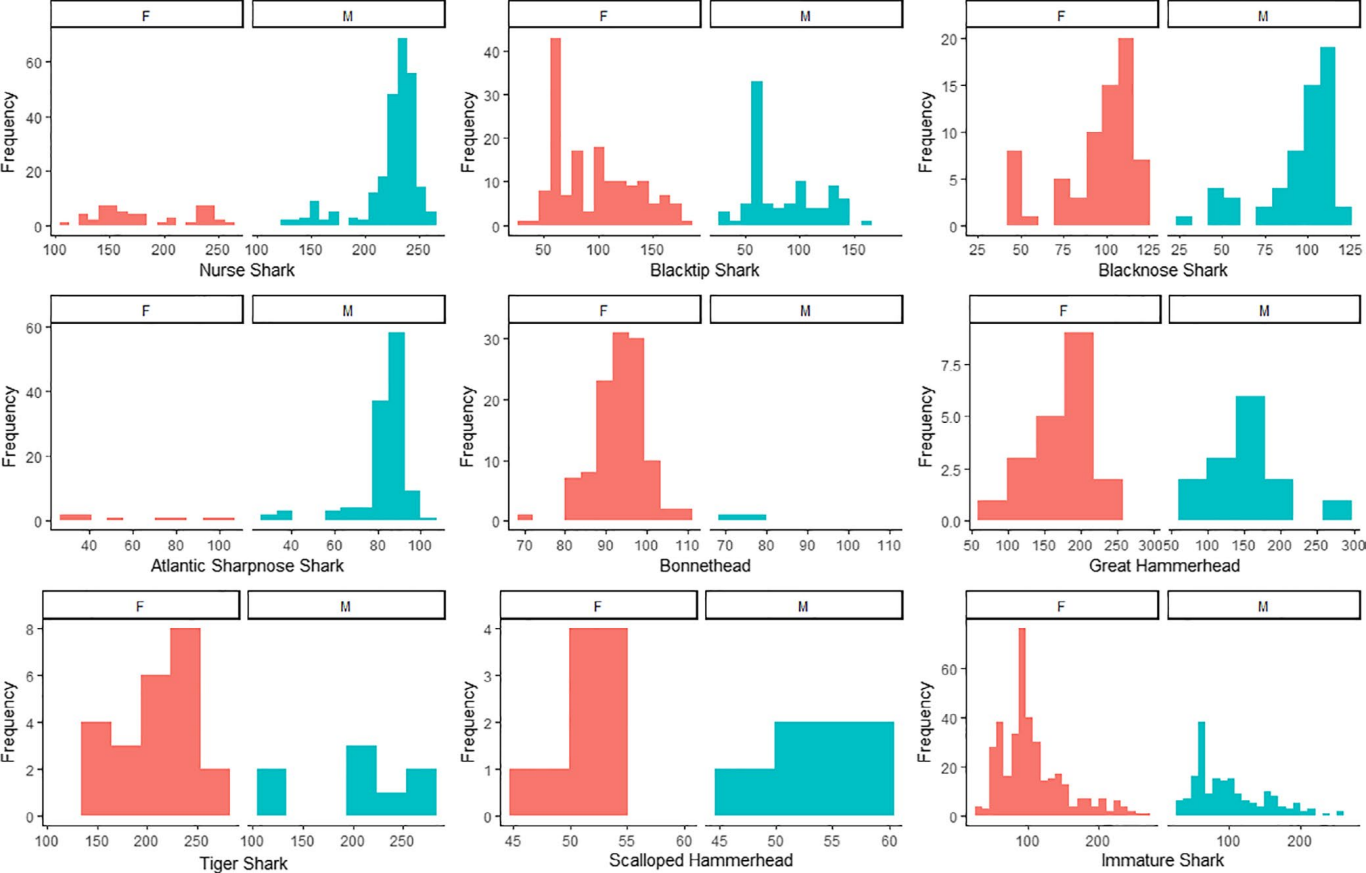
Length frequency plots (total length in cm) for subgroups with *n* > 10. Females are indicated in red and males are indicated in blue

During summer field seasons from 2013 to 2018, CMERA deployed a total of 47,833 hooks and 3090 tangle nets across 47 sites, resulting in a total of 1036 shark captures (*n*) (Table 1). Eleven different species of sharks were recorded. The five most common species had *n* > 100: nurse shark (*Ginglymostoma cirratum*), blacktip shark (*Carcharhinus limbatus)*, blacknose shark (*Carcharhinus acronotus*), Atlantic sharpnose shark (*Rhizoprionodon terraenovae*), and bonnethead (*Sphyrna tiburo*). Three species had 10 < *n* < 100: great hammerhead (*Sphyrna mokarran*), tiger shark (*Galeocerdo cuvier*), and scalloped hammerhead (*Sphyrna lewini*). Total captures slightly favored males 1.2:1; however, there was no statistical difference in sex counts across the years (*p* = .39). Two blacknose sharks and two bonnetheads were unsexed due to depredation. Of the aforementioned species, approximately half (*n* = 554) of the individuals were immature (Table 1). Size classes encompassed neonates to mature individuals (Figure 2).

**TABLE 1:** CMERA survey captures by species and maturity

Species		Total captures	Immature captures	Immature: Total ratio
*Ginglymostoma cirratum*	Nurse shark	310	69	0.22
*Carcharhinus limbatus*	Blacktip shark	245	213	0.87
*Carcharhinus acronotus*	Blacknose shark	130	93	0.72
*Rhizoprionodon terraenovae*	Atlantic sharpnose shark	130	23	0.18
*Sphyrna tiburo*	Bonnethead	120	80	0.67
*Sphyrna mokarran*	Great hammerhead	35	33	0.94
*Galeocerdo cuvier*	Tiger shark	31	31	1.00
*Sphyrna lewini*	Scalloped hammerhead	12	12	1.00
*Carcharhinus leucas*	Bull shark	10	9	0.90
*Negaprion brevirostris*	Lemon shark	8	5	0.63
*Carcharhinus isodon*	Finetooth shark	5	1	0.20
	**Total**	1036	569	0.55

Note

Immature captures were denoted according to CMERA notes and length at maturity measurements.

### 3.2 Data selection and performance

Hot spot analysis was conducted on all shark species with *n* > 10 using the Hot Spot (Getis‐Ord G*) tool in ArcGIS 10.7.1 (ESRI, 2019). Hot spot analysis was applied to CPUE values by gear type (i.e., hook or net). Sites were deemed significant at *p* < .05. Boosted regression trees were applied to species with *n* > 100. Models incorporated seven predictors (Table 2). No collinearity was present among variables except among the surface, middle, and bottom salinity parameters; due to the general mobility of coastal sharks species within the water column and the insensitivity of BRTs to multicollinearity, all three were incorporated into the model. Ultimately, salinity explained the least variation in distribution across all groups modeled (Table 3). In the final models, TD AUC and CV AUC scores were all >0.9, which suggests excellent model performance according to criteria established by Lane et al. (2009) (Table 3). Cross‐validation AUC scores were comparable to TD AUC scores, suggesting overfitting was insignificant (Hijmans & Elith, 2017).

**TABLE 2:** Source, mean values (+ SE), and range of potential predictor variables evaluated in the boosted regression trees

Predictor	Source	Mean ± SE	Range
Bottom Salinity (psu)	PCDEM	32.870 ± 0.066	25.22–36.27
Middle Salinity (psu)	PCDEM	34.295 ± 0.049	25.38–35.54
Surface Salinity (psu)	PCDEM	33.471 ± 0.048	24.39–36.17
Dissolved Oxygen (mg/L)	PCDEM	6.034 ± 0.022	4.607–8.010
Water Temperature (°C)	CMERA	29.001 ± 0.077	15.3889–33.6111
Depth (m)	CMERA	4.905 ± 0.101	0.000–15.392
Bottom type (1 = Sand; 2 = Artificial reef, 3 = Seagrass meadow)^a^	CMERA/PCDEM	N/A	N/A

^a^ Categorical variable.

**TABLE 3:** Percent contribution of each predictor output from final boosted regression trees for blacknose shark, blacktip shark, nurse shark, Atlantic sharpnose shark, bonnethead, and immature sharks

Subgroup	Training data AUC	CV AUC Score ± SE	Marginal effect 1		Marginal effect 2		Marginal effect 3	
			Variable	%	Variable	%	Variable	%
Blacknose shark	0.991	0.942 ± 0.007	Bottom Type	29.4	Temperature	24.1	DO	18.9
Blacktip shark	0.999	0.977 ± 0.005	Depth	54.4	Temperature	13.4	Bottom Type	8.2
Nurse shark	0.998	0.958 ± 0.008	Depth	28.6	Bottom Type	21.9	Temperature	17.3
Atlantic sharpnose shark	0.998	0.961 ± 0.006	Depth	29.8	Bottom Type	21.3	Temperature	17.5
Bonnethead	0.999	0.978 ± 0.007	Depth	50.3	Temperature	19.0	Middle Salinity	11.5
Immature sharks	0.998	0.975 ± 0.003	Depth	35.3	Bottom Type	22.4	Temperature	14.7

### 3.3 Nurse shark

**FIGURE 3:**
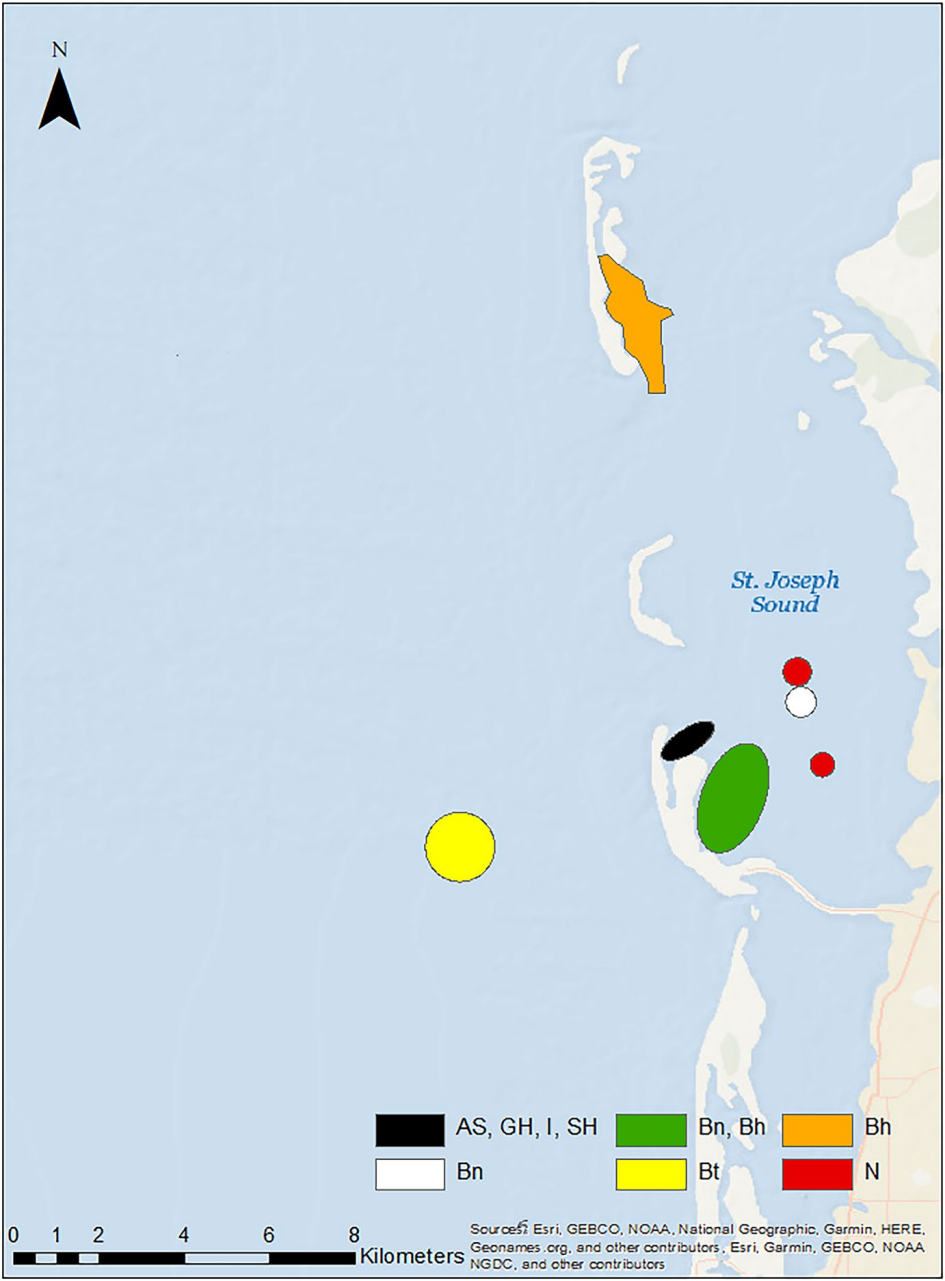
Hot spot analysis indicating areas of statistically significant clustering for subgroups of sharks captured with tangle nets. Species common names are marked as: Atlantic sharpnose shark (AS), bonnethead (Bh), blacknose shark (Bn), blacktip shark (Bt), great hammerhead (GH), immature shark (I), nurse shark (N), and scalloped hammerhead (SH)

**FIGURE 4:**
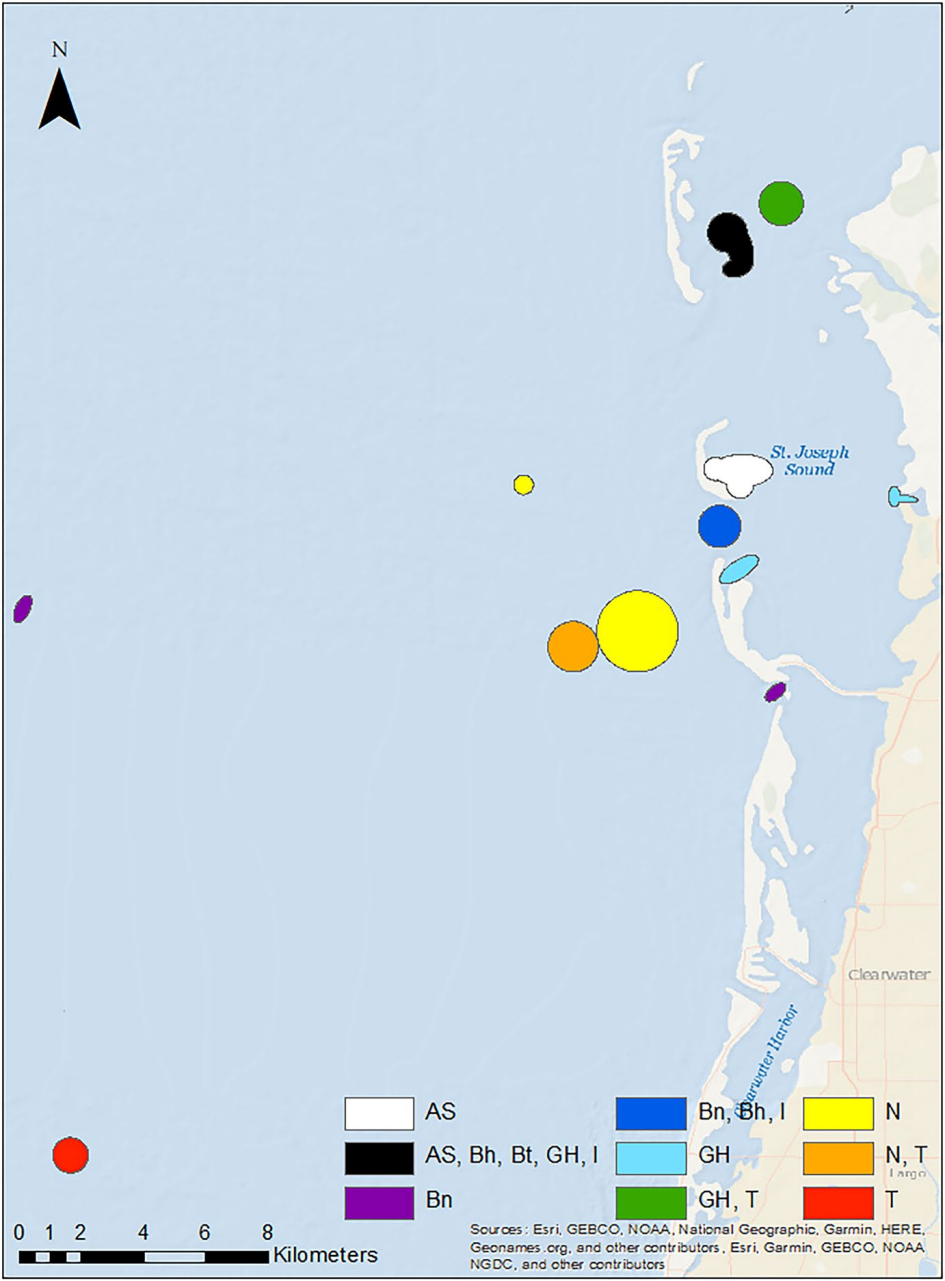
Hot spot analysis indicating areas of statistically significant clustering for subgroups of sharks caught with hooks. Species common names are marked as: Atlantic sharpnose shark (AS), bonnethead (Bh), blacknose shark (Bn), blacktip shark (Bt), great hammerhead (GH), immature shark (I), nurse shark (N), and tiger shark (T)

**FIGURE 5:**
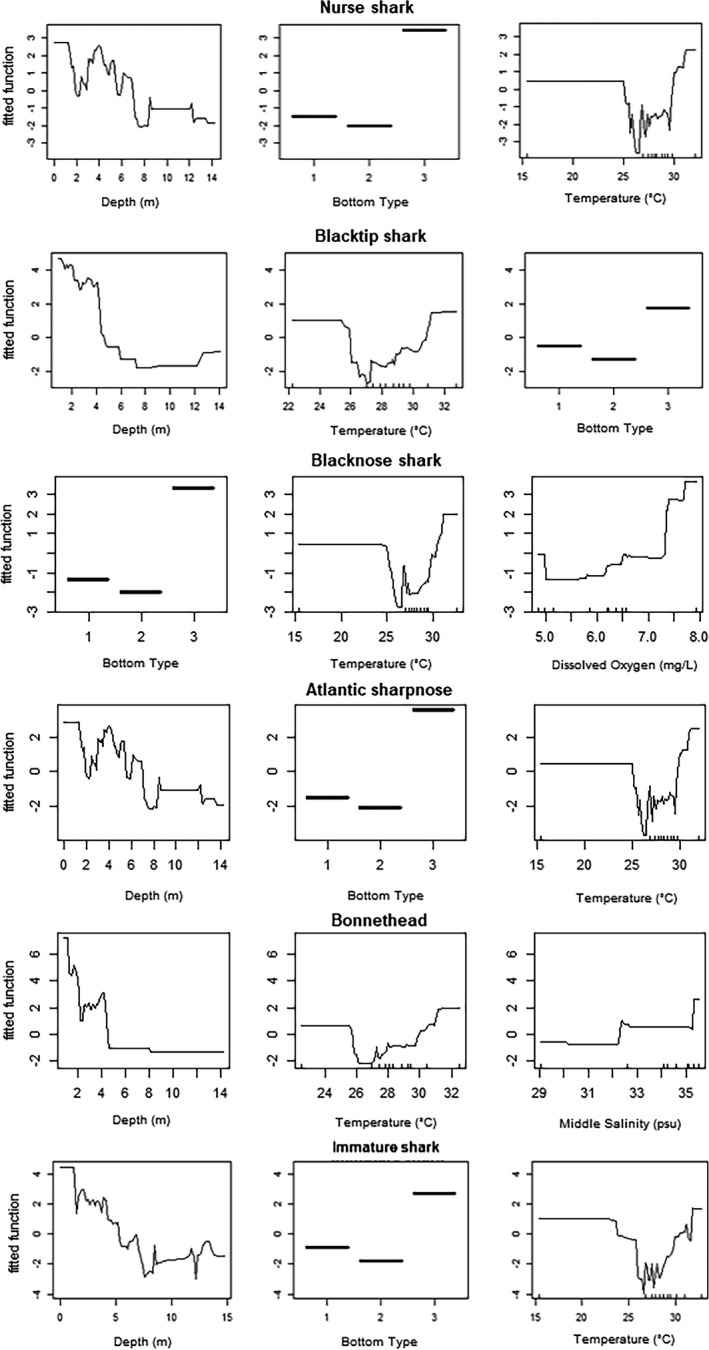
Line plots displaying the marginal effects of predictors on each subgroup (*n* > 100) derived from boosted regression trees

**FIGURE 6:**
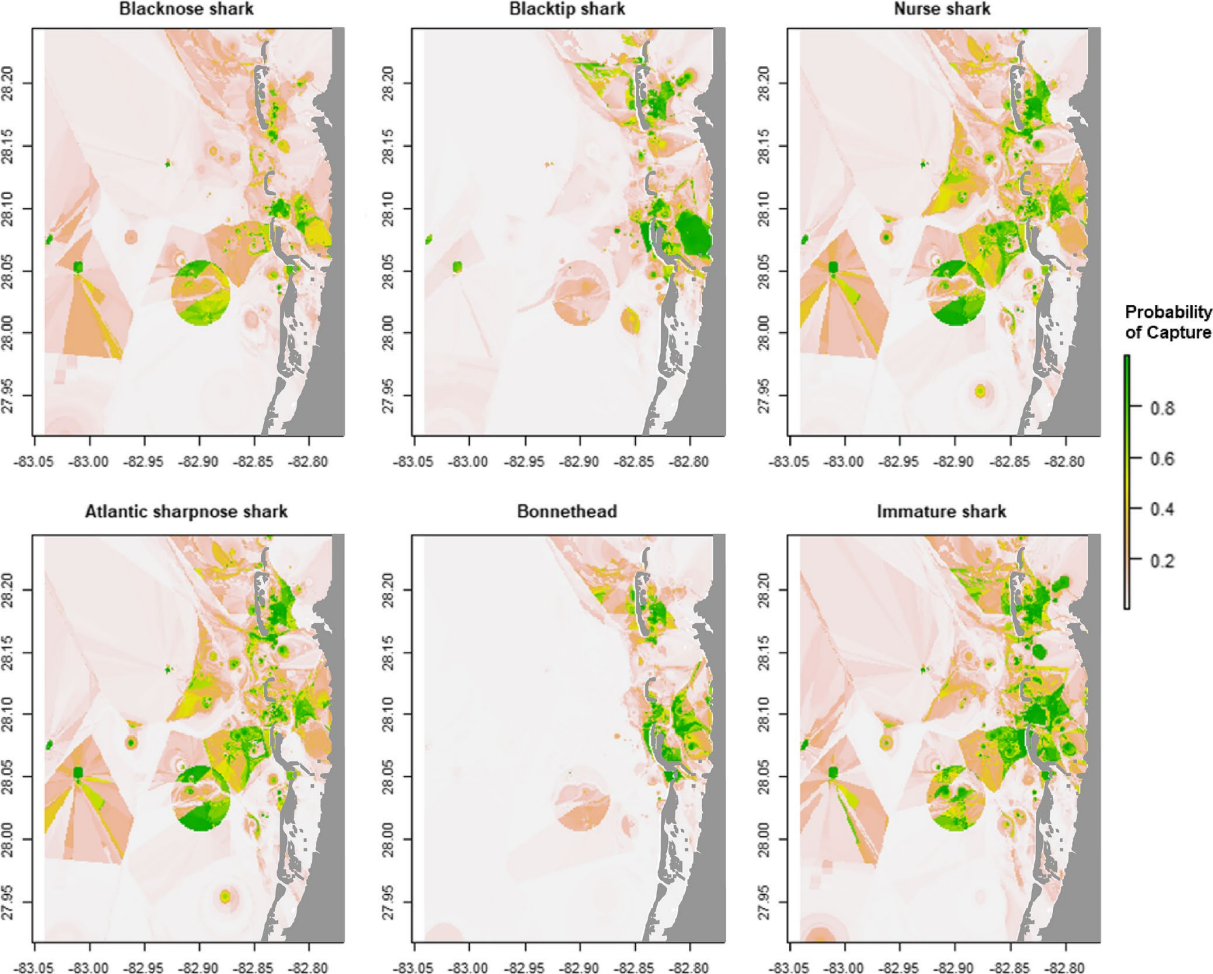
Species distribution models derived from boosted regression trees, which display suitable habitat as a proxy for probability of capture for each subgroup *n* > 100. Land is marked in grey

Nurse sharks (*n* = 310) were predominantly male (2.2:1) and were captured across a broad range of sizes (Figure 2). Nurse sharks were captured across the entire study area, but two hot spots occurred at offshore locations characterized by vegetated spoil islands and two occurred east of the barrier islands at deeper seagrass beds (Figures 3 and 4). The three most influential factors driving distribution were depth (28.6%), bottom type (21.9%), and temperature (17.3%) (Table 3). Marginal effects plots indicate a preference for >~7 m depth, seagrass bottom types, and temperatures >30℃ (Figure 5). Predicted suitable nurse shark habitat was identified at seagrass meadows surrounding the barrier islands, but also at offshore locations west of Honeymoon Island State Park and Three Rooker Island (Figure 6).

### 3.4 Blacktip shark

Blacktip sharks (*n* = 245) were predominantly female (1:1.7). Total lengths suggest the presence of both immature and mature individuals (Figure 2) according to criteria established in Baremore and Passerotti (2013). Captures occurred across the entire study area including the Intracoastal Waterway west of the barrier islands, surrounding the barrier islands, in inlets, and in St. Joseph Sound. Hot spots were located at deeper seagrass meadows located east of Anclote Key Preserve State Park and west of Honeymoon Island State Park (Figures 3 and 4). The three most influential factors influencing distribution were depth (54.4%), temperature (13.4%), and bottom type (8.2%) (Table 3). Blacktip sharks exhibited a preference for shallow depths < 4 m, temperatures > 31℃, and seagrass bottom types (Figure 5). Predicted distributions largely centered around the northern portion of Honeymoon Island State Park, nearshore habitat directly east, and in the seagrass meadows surrounding Anclote Key Preserve State Park (Figure 6).

### 3.5 Blacknose shark

The blacknose shark sample population (*n* = 130) favored females (1:1.2) with 2 unknown individuals. Between the sexes, total length distributions were similar (Figure 2). Individuals were well‐dispersed across sites, located in St. Joseph Sound, inlets, and west of the barrier islands. Blacknose shark hot spots encompassed both sandy and seagrass bottom types and were located in a variety of geographic locations (Figures 3 and 4). One hot spot occurred in the NICE zone in the seagrass meadows on the northeast side of Honeymoon Island State Park (Figure 3). Distributions were driven primarily by bottom type (29.4%), temperature (24.1%), and DO (18.9%) (Table 2). Blacknose shark preferences encompassed seagrass bottoms, temperatures greater than 30 ℃, and DO above 7.5 mg/L (Figure 5). Predicted relative abundance of blacknose sharks was highest around the southern portion of Anclote Key Preserve State Park, in the sound adjacent to it, in the tidal inlets between barrier islands, and in the sound west of Honeymoon Island State Park (Figure 6). There are also peaks of predicted relative abundance in the locations of artificial reefs and seagrass meadows west of the barrier islands (Figure 6). Hot spot locations were consistent with areas of high predicted relative abundance, while other areas of high predicted relative abundance associated with gulfside artificial reef sites were less occupied.

### 3.6 Atlantic sharpnose shark

Atlantic sharpnose shark captures (*n* = 130) were dominated by males (>13:1). Males and females were encountered across a large range of sizes, but the majority of males were mature (Table 1, Figure 2). Captures were characterized by proximity to barrier islands and occurred in St. Joseph Sound, in an inlet, and just offshore. Hot spots occurred on seagrass flats directly adjacent to the east side of Three Rooker island and east of Anclote Key Preserve State Park (Figure 4). One also occurred at the “No Internal Combustion Engine Zone” in the seagrass meadows located on northeast Honeymoon Island State Park (Figure 3). Distributions were primarily driven by depth (28.6%), bottom type (21.3%), and temperature (17.5%) (Table 3). The BRT results indicate a preference for depths at approximately 2–7 m, seagrass bottoms, and temperatures greater than 30℃ (Figure 5). Highest predicted abundance occurred east of Anclote Key Preserve State Park, near inlets, and west of Honeymoon Island State Park approximately along 28.05° latitude (Figure 6). Interestingly, the NICE hot spot on the northern end of Honeymoon Island State Park does not align with an area of most highly suitable habitat, while the other hot spots do.

### 3.7 Bonnethead

Bonnethead captures (*n* = 120) were almost entirely female (>38:1) with two unknown individuals. The three males encountered were mature, while females were both immature and mature (Figure 2) according to criteria established by Frazier et al. (2014). Captures occurred predominantly in the St. Joseph sound, in areas bordered by barrier islands. Hot spots occurred primarily in vegetated habitats. Seagrass hot spots occurred east of Anclote Key Preserve State Park and within the NICE zone on the northeast side of Honeymoon Island State Park (Figures 3 and 4). One hot spot, located east of Anclote Key Preserve State Park, was characterized by both seagrass flats and mangroves (Figure 3). They also occurred in the sandy inlet between Three Rooker Island and Honeymoon Island State Park (Figure 4). Bonnethead distribution was driven by depth (50.3%), temperature (19.2%), and middle salinity (11.5%) (Table 3). They preferred depths < 4 m, temperatures > 30℃, and salinities > 32 psu (Figure 5). The geographic extent of predicted suitable habitat was smaller than the other subgroups evaluated, likely due to the dominant influence of depth and lesser influence of bottom type (Figure 6). Suitable habitat largely encompassed nearshore, barrier island habitat, which aligns with the locations of their hot spots.

### 3.8 Immature shark

Immature sharks (*n* = 569) were identified among the sample populations of each species with *n* > 10 (Table 1). Female immature sharks outnumbered males 2:1. In particular, all scalloped hammerheads and tiger sharks were immature, and immature individuals comprised most captures (>50%) for blacknose shark, blacktip shark, and bonnethead (Table 1). Immature sharks were present across the study area. Interestingly, two of the three identified hotspots were in or adjacent to the NICE zone (Figures 3 and 4). All hot spot locations were characterized by their close proximity to the barrier islands: a seagrass flat east of Anclote Key Preserve State Park, a sandy inlet between Anclote Key Preserve State Park and Honeymoon Island, and seagrass bottom on the northeast side of Honeymoon Island (Figures 3 and 4). For immature sharks, depth (35.3%), bottom type (22.4%), and temperature (14.7%) were the three most influential factors influencing distribution (Table 3). Immature sharks showed a preference for depths < 5 m, seagrass bottom types, and temperatures >~30℃ (Figure 5). Their predicted distribution displayed peak predicted relative abundance surrounding the barrier islands and at seagrass meadows located east of the barrier islands, with other peaks in predicted abundance occurring at locations of artificial reefs further offshore (Figure 6).

### 3.9 Great hammerhead, Tiger shark, and Scalloped hammerhead

Great hammerhead captures (*n* = 35) were dominated by females 1.5:1 and occurred primarily within the bounds of the barrier islands in St. Joseph Sound. Total length distributions were similar between sexes (Figure 2). Nearly two‐thirds of the captures were immature individuals (Table 1). Great hammerhead hot spots included the seagrass bottom NICE zones on the northeast side of Honeymoon Island State Park for both gear types, two seagrass flats east of Anclote Key Preserve State Park, and a small nearshore seagrass flat surrounded by mangroves (Figures 3 and 4).

Tiger sharks (*n* = 31) were predominantly female (1.5:1) and immature (Table 1) based on criteria set forth by Kneebone et al. (2008). In contrast to other subgroups in this study, the majority of individuals were captured west of the barrier islands. Tiger shark hot spots occurred offshore at an artificial reef location, a deeper seagrass bed on the Gulf side of Honeymoon Island State Park, and at a deeper seagrass flat east of Anclote Key Preserve State Park in St. Joseph Sound (Figures 3 and 4).

Scalloped hammerhead captures (*n* = 12) were equally distributed between the sexes and entirely immature (Table 1) according to Castro (2010). They were only first observed beginning in 2017. Captures were rare across the study area and only occurred in seagrass locations in the NICE zone. The sole scalloped hammerhead hot spot was located in the NICE zone on the northeast side of Honeymoon Island State Park (Figure 3).

## 4 DISCUSSION

A diverse assemblage of sharks was observed by CMERA volunteers. Eleven species were identified, with five species dominating shark abundance: nurse shark, blacktip shark, Atlantic sharpnose shark, blacknose shark, and bonnethead. The diversity of species present in the study area, as well as the diversity in sex and size, suggests co‐residency of multiple species surrounding the barrier islands; however, the unique distribution and abundance of each species within the study area suggest species‐specific partitioning of habitat within the relatively small area. These results are representative of late spring and summer spatiotemporal distributions across 2013–2018. Given that many of the species exhibit reproductive behaviors during this seasonal time frame, it is likely that these distributions are unique only to late spring and summer and should not be extrapolated to fall, winter, and early spring. Other studies have provided insight into shark habitat use and partitioning within the Gulf of Mexico (Bethea et al., 2014; Drymon et al., 2020; Froeschke et al., 2010) and oceanwide (Brodie et al., 2015; Santos & Coehlo, 2018). This study is unique in that it provided the opportunity to identify and quantify factors correlated with shark distribution on a smaller scale, using unconventional data sourced from a research and education program.

Peterson and Grubbs (2020) evaluated shark abundances in the coastal waters of the Florida Big Bend, a region just north of this study site that is similarly dominated by seagrass habitat and low riverine input. Their species diversity largely mirrored that identified in this study, with the exception of the scalloped hammerhead uniquely identified in these west‐central coastal waters. Great hammerhead populations were comparable between the two locations. In addition, both assemblages were composed of majority immature sharks. Despite similar species composition, the regions were marked by species‐specific differences in abundance and life stage. Nurse shark CPUE values were twice those reported in the Big Bend, and juveniles were nearly three times as abundant. Blacktip shark CPUE was approximately five times less than in Big Bend; however, sex and size ratios were comparable. Relative abundances of blacknose sharks between the Big Bend and west‐central Florida were comparably dominated by immature individuals (Peterson & Grubbs, 2020). These results align with the Hueter and Tyminski’s (2007) theory that both regions may be providing blacknose shark nursery habitat.

Despite many similarities, there were pronounced differences between the assemblage characterized in the current study and the one caught in Peterson and Grubbs (2020). Peterson and Grubbs (2020) caught an order of magnitude more Atlantic sharpnose sharks and had nearly quadruple the proportion of immature captures. Given that Atlantic sharpnose sharks are not known to use nursery habitat, investigation into the stark difference in immature populations along the longitudinal gradient may be merited. In contrast, the relative abundance of bonnetheads in west‐central Florida was as much as an order of magnitude larger than in the Big Bend (Peterson & Grubbs, 2020). The proportion of immature bonnethead captures were comparable; however, this study's bonnethead sample was strongly dominated by females as opposed to the male‐dominated sample in the Big Bend. Given that mature female bonnetheads are known to use nearshore areas for gestation and pupping (Driggers et al., 2014), the sexual segregation may suggest that this west‐central region may be an important habitat for bonnethead reproduction.

While these regions are characterized similarly by heterogeneous bottom types with high seagrass coverage, low energy systems, and low riverine input, our sampling site is unique in that it is also heavily influenced by the presence of barrier islands, which may explain some of this variation in species abundance and life‐history composition. Bethea et al. (2014) noted greater shark species diversity associated with barrier islands near riverine‐influenced systems in the northern Gulf of Mexico. In the absence of highly variable salinity associated with riverine input, the effects of the barrier islands are more pronounced in this study, and the benefits they provide (e.g., a physical barrier from larger predators in the Gulf) may explain why species characterized by nearshore nursery use and site fidelity, such as the bonnethead (Heupel et al., 2006) or nurse shark (Castro, 2000), have higher immature abundances in west‐central Florida. Further, the results from the Big Bend contrast to this region's lesser abundance of immature Atlantic sharpnose sharks, whose life‐history strategy would not benefit as much from enhanced nursery habitat provided by barrier islands. These clear differences in species composition and life stages demonstrate longitudinal variation in the composition of coastal shark populations, potentially due to the influence of barrier islands, despite being located in otherwise similarly characterized environments.

Sex ratios varied by species, with the most extreme segregation apparent in the Atlantic sharpnose shark and bonnethead. Consistent with the findings of Parsons and Hoffmayer (2005) and Drymon et al. (2020) in the northern Gulf of Mexico, the shallow waters of west‐central Florida were largely dominated by adult male Atlantic sharpnose sharks, supporting the notion that following maturation, females remain predominantly offshore, where both maturation and pupping occur. While Atlantic sharpnose sharks are known to spatially overlap at immature and mature life stages, immature individuals are highly mobile in coastal habitats and are not known to depend heavily on nursery areas (Heupel et al., 2018), a finding further corroborated by this study's largely adult male sample. While the prevalence of adult males nearshore contrasts with the explanation proposed by Parsons and Hoffmayer (2005), who suggest that males may migrate offshore for mating during summer in the northern Gulf of Mexico, Drymon et al. (2020) found that male sharpnose sharks can be prevalent both inshore and offshore during the summer. As a result, the mating season of Atlantic sharpnose sharks in the eastern gulf may not overlap spatially or temporally with the northern gulf, indicating geographic variability which aligns with the findings of Hoffmayer et al. (2013). In contrast to the Atlantic sharpnose, the bonnethead population was largely dominated by females. These findings are consistent with a survey of sharks in shallow South Carolina waters, which found that the nearshore bonnethead population was largely pregnant females (Ulrich et al., 2007). Bonnetheads are small coastal sharks whose females do not use estuaries for nursery habitat, but rather move offshore in late summer for parturition and mating (Driggers et al., 2014). Rather, it is thought that gravid females utilizing estuarine habitat are taking advantage of the availability of high energy benthic prey prior to parturition in August in order to decrease the gestational period, which is one of the shortest compared to other species of sharks (Manire et al., 1995). The sexual segregation of the shark population in this region suggests west‐central Florida may not be critical reproductive habitat for the Atlantic sharpnose shark, but may function in this capacity for bonnetheads.

The results of hot spot analyses indicate interesting spatial variation in habitat use among species within a relatively small study area. The magnitude of species utilizing the NICE zone in statistically significant abundances (i.e., as a hot spot) points to the potential ecological significance of this management practice. Given that seven different subgroups are highly concentrated in this zone, the benefits of reduced motorized boating activity may be outweighing the cost of interspecific competition. In the instance of the Atlantic sharpnose shark, utilization of the NICE zone as a hot spot outweighed the BRT’s identification of most highly suitable habitat, as the two did not align for this species. Other hot spots were generally centered around seagrass habitats near the barrier islands and were unique to each species. For captures resulting from longline fishing, a greater majority of hot spots occurred offshore, particularly for larger species such as the nurse shark, which were largely mature, and the tiger shark, which were largely immature. Interestingly, the tidal inlet between Three Rooker Island and Honeymoon Island State Park served as a hot spot for blacknose sharks, bonnetheads, and immature sharks, indicating it may serve as a geographic bottleneck for individuals moving between St. Joseph Sound and the Gulf of Mexico. It is possible this hot spot may also exist due to its proximity to the NICE zone and the geographic protections offered by the shape of northern Honeymoon Island. Another significant hot spot of high species diversity was identified east of Anclote Key Preserve State Park, an area described as relatively deep seagrass flats. This hot spot further underscores the importance of seagrass habitat and barrier islands as critical habitat for sharks of many species and life stages.

Hot spots can be used to guide management decisions for recreational fishing in the area. Oceanwide studies have identified the threat to shark populations caused by overlap between hot spots of pelagic shark abundance and fishing hot spots (Queiroz et al., 2019). Given that Florida is a hot spot for shark fishing in the United States (Shiffman & Hammerschlag, 2014), careful consideration should be taken to avoid unsustainable shark fishing practices in coastal hot spots as well, particularly for species that are protected or are using the area for reproduction. Currently, Florida law 68B‐44.004 identifies great hammerhead, lemon shark, scalloped hammerhead, and tiger shark as prohibited species and grants them special protections. Despite these protections, post‐release mortality rates can still be high, particularly for the two hammerhead species (Dapp et al., 2016; Gallagher et al., 2017). Many of the hot spots for the shark population of this study area surround barrier islands, where mooring is permitted and land‐based fishing may pose a threat. To maintain healthy status of non‐protected species, fishing of immature and protected individuals in the area should be monitored closely and perhaps prohibited where especially vulnerable species, such as hammerheads, are known to aggregate.

The preponderance of immature individuals in this study suggests the potential of this area as a nursery habitat. More than half of all captures in this study were immature sharks, and for many species, such as the blacknose shark, blacktip shark, bonnethead, great hammerhead, scalloped hammerhead and tiger shark, immature sharks were the vast majority of captures. In the northern Gulf of Mexico, the blacknose shark assemblage is dominated by mature individuals (Drymon et al., 2020), yet the abundance of immature blacknose sharks in the current study suggests the shallow waters of west‐central may function as blacknose shark nursery habitat. Hueter and Tyminski (2007) have identified the gulf coast of Florida as nursery habitat for blacknose, blacktip, and great hammerheads, which is consistent with our predominantly immature sampling of these species. Although we identified a majority immature bonnethead population, bonnetheads do not necessarily utilize nearshore habitat as nursery sites. Rather, young‐of‐year may move into estuaries and nearshore habitat to reduce predation risk from deeper waters, which may explain the large proportion of immature bonnetheads in this study area (Swift & Portnoy, 2020). While capture numbers were relatively low, nearly all the tiger sharks and all scalloped hammerheads encountered were immature, suggesting future work should focus on quantifying the importance of this region as critical habitat for these species, particularly since scalloped hammerheads have been previously noted as uncommon in these waters (Hueter & Tyminski, 2007). The significant proportions of immature sharks in this study area certainly merit further research to allow this area to be evaluated according to the criteria established by Heupel et al. (2007).

The impacts of anthropogenic stressors on shark nursery areas can be difficult to quantify (Ward‐Paige et al., 2015). However, as human population densities within 100 km of the ocean exceed triple the global mean, it is crucial to understand these impacts on wildlife, particularly near coastal metropolitan areas such as those examined in this study (reviewed in Whitfield & Becker, 2014). The results of the BRT indicate nearshore coastal areas characterized by shallow, warm, seagrass environments provide critical habitat for shark species and immature sharks in general. In context, shallow depths and warmer waters can be conflated simply as characteristic of seagrass habitat which is associated with greater prey availability for predatory fishes, even when compared with nearby unvegetated habitats (Rozas & Odum, 1988). By underscoring the importance of these nearshore habitats, it is also important to consider that some of these species use offshore environments in different life stages or as segregated groups (Drymon et al., 2020; Parsons & Hoffmayer, 2005) and that damage felt in nearshore environments, such as habitat loss or poor water quality, may not only affect these coastal populations, but can also have ripple effects for offshore populations. While the barrier islands in this study are state parks and thus protected from development, they are still visited by tourists and subject to boating traffic, mooring, and land‐based fishing. Given the dense human coastal population and the draw of the barrier islands to visitors, it is essential that these locations are properly managed. Because of the pressures of human activity in these essential nearshore environments, these critical nearshore habitats identified by the BRT require careful protection and management in order to support healthy shark populations.

While providing spatially explicit recommendations for future management efforts, this study also offers insight into currently enacted management practices. As demonstrated by the diversity of species hot spots located within the NICE zones, it appears that reduced threats from boating activity may be fostering suitable habitat for sharks. Management in the form of NOAA’s marine protected areas (MPAs), which largely regulate public access and activities, has been shown to increase fish abundance in the coastal waters of Florida (Bohnsack, 2011) and in other tropical regions (Bond et al., 2012). Strikes from boat propellers may be fatal, but sites subject to boat wakes have also been associated with lower levels of faunal abundance and diversity, as well as destruction of essential seagrass habitat (Whitfield & Becker, 2014). Immature individuals are particularly vulnerable to boat strikes, and a loss of seagrass structures would likely result in a decrease in prey availability and a loss of three‐dimensional structure in which immature individuals find protection from predators. The northern end of the NICE zone is located near an inlet, which provides access from St. Joseph Sound to the Gulf of Mexico. These inlets between the islands may create a geographic bottleneck for sharks and other highly mobile migratory animals, including potential shark prey, between the Gulf and St. Joseph sound. This location is an example where a hot spot for sharks may also be a hot spot for boating activity. The link between NICE zones and a higher abundance of sharks, immature sharks in particular, should be used to advocate for expansion of NICE zones to areas of suitable habitat for Florida‐protected sharks currently utilizing these zones (i.e., great hammerhead and scalloped hammerhead).

These spatial, quantitative, and qualitative insights were made possible by data collected through a research and education program. In a field where baseline abundance data are often lacking for conservation and management interests (Ward‐Paige & Worm, 2017), researchers have been criticized for failing to use pre‐existing datasets collected outside of academia (Buxton et al., 2021). Acknowledging that the uneven sampling effort and haphazard site selection in this study limits some analyses, this work demonstrates the capacity of a limited dataset, collected through a private citizen research and education group, to provide useful information for management. In particular, these results can be used to direct spatial prioritization of management practices as well as identify the common traits that characterize these areas (e.g., proximity to barrier islands, bottom type, NICE zones) for extrapolation outside of the study area. Future efforts to characterize population trends from unconventional data should consider using BRTs. Boosted regression trees are an ideal tool for analysis of incomplete datasets, as they can be tailored to presence/absence values and are robust to missing values, outliers, and multicollinearity (Dedman et al., 2017). Through the application of these BRTs, we were able to overcome the limitations imposed by haphazard site sampling, and extrapolate to identify predicted relative abundance across the study area during the summer. This work exemplifies the capacity of datasets sourced from extra‐academic sources to provide meaningful fisheries management information given careful and appropriate selection of analyses. With this kind of work, scientists can resourcefully provide the supporting information needed to guide successful management of fisheries.

## CONFLICT OF INTEREST

None declared.

## AUTHOR CONTRIBUTIONS

**Lindsay L. Mullins:** Conceptualization (lead); Data curation (lead); Formal analysis (lead); Investigation (lead); Visualization (lead); Writing‐original draft (lead); Writing‐review & editing (lead). **J. Marcus Drymon:** Conceptualization (equal); Formal analysis (equal); Investigation (equal); Resources (equal); Supervision (equal); Writing‐review & editing (equal). **Moriah Moore:** Resources (equal); Writing‐review & editing (supporting). **Adam Skarke:** Supervision (equal); Writing‐review & editing (equal). **Alan Moore:** Resources (equal); Writing‐review & editing (supporting). **John C. Rodgers:** Conceptualization (equal); Resources (equal); Supervision (equal); Writing‐review & editing (equal).

## Data Availability

All data associated with this publication (capture and environmental data) can be accessed on Dryad (https://doi.org/10.5061/dryad.b2rbnzsgc).
